# Supplementary material for: Attentional focus strategies in racket sports: A systematic review
Source: PLoS One. 2024 Jan 5;19(1):e0285239. doi: 10.1371/journal.pone.0285239 (PMC10769023; doi:10.1371/journal.pone.0285239)
Supplement: Attachment — Submitted filename: Response to Reviewers.docx [file pone.0285239.s003.docx]

**RESPONSE TO REVIEWERS COMMENTS**

**Dear Editor and Reviewers,**

**The authors would like to thank the Reviewers for the opportunity to improve our manuscript for the second time. All requested changes have been included in the revised paper and highlighted in yellow. Likewise, we hope that our modifications and explanations meet your expectations. We feel our revised manuscript is much improved.**

**Reviewer #1**

Minor Comments

1. Pg. 3 Lines 66-67: Is the opponent’s side of the table also referred to as “the opponent’s court” in the racket sport of table tennis?

**Response: We have rephrased this sentence for clarity.**

2. Pg. 7 Line 162: Do you mean “≥6”?

**Response: This has been changed.**

3. Pg. 9 Line 212: Check the expression in “Three of the nine…their sample size” so that its intended meaning is clear.

**Response: We have rephrased this sentence for clarity.**

4. Pg. 9 Line 214: Remove the comma after the brackets.

**Response: This has been changed.**

5. Pg. 11 Table 2: Check the consistency in using upper- and/or lower-case letters for the column headings.

**Response: This has been changed.**

6. Pg. 13 Table Footnote: Check the consistency in using hyphens or em dashes.
**Response: This has been changed.**

**Reviewer #2**

I would like to thank the authors for addressing most of my comments. For future submissions, I would like to advise the authors to include textual changes made as part of their response, as that will make it easier to review (and avoids any potential misinterpretations as part of this process).
**Response: We would like to thank the Reviewer for the thoughtful comments that were provided on the previous version of this paper. We have revised the requested sections according to the above suggestions. Please see the highlighted and indicated text in the lines.**

I have a few additional suggestions/comments, especially with regard to the discussion and conclusion section:

- Discussion - page 16, lines 320-321: “It has been noted that the advantages of external focus bring about a variety of effects that we cannot fully comprehend. On average, however, these effects tend to be minimal or even non-existent [42].”
It is not clear from this sentence what the advantages are that we cannot fully comprehend or why they appear to be minimal or non-existent. Either clarify this further or consider removing altogether.

**Response: We have rephrased these sentences for better clarity, as shown in lines 321–328.**

- Discussion – page 16, lines 322-327: It is stated that the findings presented here support the predictions of the constrained action hypothesis, but that seems to be overinterpreting the current results. That is, the constrained action hypothesis specifically predicts that movement performance is enhanced through increased automaticity, but based on the results presented it seems impossible to make any conclusions as to whether those that received an external focus indeed moved more automatically (this would require assessments of outcomes indicative of automaticity, which are not really presented here).

**Response: We agree with the comment, and we have addressed the concern mentioned by removing the statements on the constrained action hypothesis and have rephrased the paragraph to improve clarity and understanding, as presented in lines 329–341.**

- Discussion – page 17,18, lines 367-373: I like the additional discussion on the potential benefit of proximal EF for novices here. The final conclusion presented here regarding skill differences and proximal/distal EF seems to be inconsistent with the information presented before though: Based on the findings in the review, is the main conclusion that skill level does not make a difference (and that both proximal and distal EF would be equally effective)? If so then please clarify why this is concluded, as I wonder if the evidence presented in the review sufficiently strong to make this claim?

**Response: We agree with the reviewer, and we have revised the paragraph to address the inconsistency noted by the reviewer, as shown in lines 371–388.**

- I think a key limitation of the reviewed studies (as with most studies in motor learning) is the limited follow-up period. As skill acquisition in practice typically unfolds at far longer time scales, we have to acknowledge that there is inherently limited scope to generalise results from studies like the ones reviewed here to real-world application. It would be good to discuss this explicitly in the review’s discussion.
**Response: We appreciate your comments and suggestions. We have included further discussion in the revised version of this paper, as shown in Lines 451–463.**

- Given the limited evidence available for the use of holistic cues or similar, I would remove reference to these / the recommendation to explore these from the main conclusion section within the discussion (and just discuss this only as part of the rest of the main text, as potential avenue for research and application).

**Response: We addressed the issues by relocating the recommendations about holistic focus and analogies from the conclusions section to the discussion, as demonstrated in Lines 417–19, as shown in Lines 417–419.**
